# Test-retest reliability of postural control measures in healthy secondary school pupils: insights from the MOVE12 pilot study

**DOI:** 10.3389/fspor.2025.1521252

**Published:** 2025-01-23

**Authors:** Sigbjørn Litleskare, Svein Barene

**Affiliations:** ^1^ Department of Public Health and Sport Sciences, University of Inland Norway, Elverum, Norway; ^2^ Centre for Health and Technology, University of South-Eastern Norway, Drammen, Norway

**Keywords:** postural stability, postural instability, postural sway, centre of pressure, centre of gravity

## Abstract

**Introduction:**

Postural control is an essential part of human movement. Measurements of center of pressure displacements by force platform are considered the gold standard for assessing postural control. However, the test-retest reliability of these measurements in field-based conditions is unclear.

**Methods:**

This study aimed to investigate the test-retest reliability of center of pressure measures under field-based conditions, and assess the impact of height, weight, and body mass index (BMI) on test-retest reliability. The study sample comprised 215 upper secondary school pupils (114 girls) aged 16–17 years, all drawn from the control group of a larger intervention study. There was a 12-week interval between the initial test and the retest. Measurements of center of pressure displacements was assessed during a bipedal eyes closed and a unipedal eyes open condition. Interclass correlation coefficients (ICCs) with 95% confidence intervals were calculated for six distinct measures in both conditions. Correlations between the change in postural control and height, weight, and BMI were assessed as well.

**Results:**

Trace length exhibited the best test-retest reliability in both conditions, achieving moderate to good reliability in the bipedal eyes closed condition (ICC = 0.73, 95% CI = 0.66–0.78). Test-retest reliability was higher for all measures in the bipedal eyes closed (ICC's = 0.53–0.73) compared to unipedal eyes open condition, and some of these measures approached zero reliability (ICC's = 0.03–0.48). Among body characteristics, only height was significantly correlated with test-retest changes in postural control (*r* = 0.16, *p* < 0.05) and only for two of the measures.

**Discussion:**

This study underscores the necessity of careful selection of postural control measures and thorough assessments in field-based research to achieve acceptable test-retest reliability.

## Introduction

Almost all of our daily tasks require some ability to maintain an upright standing position and to control our posture while standing (1, 2). This ability, often referred to as postural control, relies on the capacity to accurately perceive the body position in relation to the surroundings through vestibular, visual, and proprioceptive inputs and adjusting posture according to these inputs (3). Measurements of center of pressure (COP) displacements by force platform are considered the gold standard for assessing postural control under static conditions, and a large number of COP measures have been proposed (4–7). However, there is disagreement regarding the most appropriate and reliable measures (8), which has been documented through long-standing discussions in the scientific community (7, 9, 10).

Test-retest reliability studies within this field typically comprise limited sample sizes, individuals with existing health conditions limiting heterogeneity, and are usually carried out under highly controlled conditions with two or more consecutive attempts per participant to increase reliability (8, 11–16). Previous research often report different examination conditions that affect reliability of postural stability parameters, such as open and closed eyes (17), visual target distance (18), and feet position (19). According to a previous systematic review of COP test-retest reliability by Ruhe et al. (5), it is concluded that with sufficient number of repetitions and sampling durations, all COP parameters will achieve acceptable reliability. Thus, the question is not whether reliable measures can be obtained, but rather if reliable measures can be obtained in typical test situations, such as field-based testing of large groups of diverse individuals. During field-based mass-testing, less controlled conditions can be expected, and due to time constraints, multiple attempts per participant are not feasible. The test-retest reliability of COP measures under these conditions is therefore largely unknown.

Sample size in a test-retest study affects the level of accuracy of reliability estimates. The general advice is to ensure a sample size of at least 50 participants, while a sample size of 200 is preferable (20). Sampling issues are also a concern since homogeneity may lead to reduced reliability estimates (20, 21). Researchers conducting a test-retest study should ensure that population heterogeneity is obtained, preferably through random sampling to ensure a diverse range of participants in relation to the outcome being investigated (e.g., postural control). In cases where random sampling is not feasible, other measures should be implemented to increase heterogeneity, such as multi-site sampling (20). Concurrently, the sample should be described in sufficient detail to allow conclusions about its heterogeneity (20). In their review, Ruhe et al. (5) reported that approximately 30% of their included studies either inadequately described participant selection criteria or provided no description at all, while this information was adequately reported by approximately half of the studies. Ruhe et al. (5) further revealed that some of the inter-subject variability observed in their reviewed studies could be attributed to the learning effect, and that differences between within-day trials resulted in higher test-retest reliability estimates compared to between-day trials. Thus, retests should be performed with a sufficient wash-out period to avoid a potential learning effect (8, 22). Although previous research suggests that height, weight, and body mass index (BMI) negatively impact postural control (8), the impact of these factors on test-retest reliability is poorly understood and underreported Research suggests that height may impair postural control due to longer neural signal paths and increased distance between the center of gravity and the base of support (8, 23). Additionally, increased weight and BMI has been associated with reduced postural control, possible because of a more anterior center of gravity and potential desensitization of mechanoreceptors (5). These anthropometric factors may lead to more variable performances, negatively impacting the test-retest reliability of postural assessments. Ruhe et al. (5) emphasizes the importance of documenting these effects in reliability studies.

Despite the previously mentioned limitations of research within this field, traditional measures, such as distance measures (trace length/sway path length), have previously been reported to be the among most reliable measures of postural control (5). Newer methods have been proposed to improve upon these traditional measures (24, 25), but results generally lack independent confirmatory replication (8, 26). In terms of experimental conditions, previous research reports that eyes closed conditions tend to generate the most reliable results (8), while maintaining postural control on one foot may be considered more challenging and, thus, more sensitive to intervention effects (27).

The aim of this study was to assess test-retest reliability of different COP measures with an adequate sample size under field-based conditions. The hypotheses were:

I. Total trace length of COP will display the highest level of test-retest reliability.
II. A bipedal eyes closed condition will lead to higher test-retest reliability compared to an unipedal eyes open condition.
III. Height and weight will be negatively associated with the test-retest reliability of COP measures.

## Materials and methods

### Study design

This test-retest reliability study was carried out as a part of a 12-week randomized controlled physical activity pilot study among Norwegian upper secondary school pupils between January and May 2023. The inclusion criteria for participation in the study were pupils aged 16–17 years. Exclusion criteria were defined as specific disabilities that make participation impossible and/or specific illnesses that can cause health hazards, e.g., ankle fractures, cerebral palsy and/or undefined disabilities that clearly posed challenges to completing the tests in accordance with protocol. For more information regarding the MOVE 12 pilot please refer International Standard Randomized Controlled Trial Number Register (ISRCTN10405415). The study was approved by the Research Ethics Committee at Inland Norway University of Applied Sciences, Norway (21/01894), and registered in the International Standard Randomized Controlled Trial Number Register (ISRCTN10405415). All participants gave their written informed consent to participate in the study.

### Participants

The sample used in this reliability study comprised the 215 pupils (114 girls) in the control group to adhere to general recommendations of including ≥200 participants in test-retest studies (16). The overall aim of the pilot study was to ensure a 50/50 distribution from schools offering educational programs for specialization in general studies and vocational study programs, respectively. Among them, half would be randomly assigned to the control group and included in the reliability study. Invitations to participate were distributed to all 25 upper secondary schools in the municipal county of Viken, of which 5 schools were selected based on a combination of the stratification and convenience principle. In consideration of that stratification principle, we wanted a certain geographical spread in the schools, while for reasons of convenience we chose to recruit a double number of classes/pupils from the largest school with a vocational study program. This resulted in a total of five schools being included in the study, i.e., three with education program for specialization in general studies (80–100 pupils per school) and two schools with vocational study programs (80–100 at one school and 160–200 at the other school). The participating pupils were instructed to maintain their regular level of physical activity throughout the study period.

### Randomization

As mentioned above, participants were randomly assigned to either the intervention or the control group. The randomization was made by lot by blinded staff, i.e., classes were assigned to either an intervention group or a control group (1:1 ratio). At each of the three schools consisting of classes (*n* = 25–30) with specialization in general studies, the school management initially selected four classes/groups with the most homogeneous characteristics possible. The selection was conducted by drawing from two boxes: (i) the two different groups (the intervention group or the control group) and (ii) the four classes/groups (1, 2, 3 or 4). The process was initiated by drawing one group from box 1, followed by drawing one class/group from box 2. The next draw from box 2 was consequently allocated to the remaining group in box 1. This process was repeated until all the classes had been assigned to either an intervention group or the control group. At the two schools that consist of vocational study programs with smaller class sizes (*n* = 12–17), stratification was carried out in collaboration with the school management with the aim of matching classes/groups on gender, number of pupils, and subject area, respectively, which were then distributed in four separate boxes (box A, B, C or D). The selection was initiated by drawing a group from box 1 (the intervention group or the control group), followed by drawing a class/group from one of the A-D-boxes. This process was repeated until all classes had been distributed equally to the intervention groups and the control group, respectively.

### Instruments

The participants' postural control ability was assessed using a force platform (FP 4, Hur labs Oy, Tampere, Finland) and the associated software (HUR Labs Force Platform Software Suite). One sensor is placed in each corner of the platform and these sensors are capable of measuring weights up to 200 kg. The platform is equipped with a 16-bit data acquisition module, which includes an integrated analog-to-digital (a/d) conversion. To prevent any potential interference or cross talk, a separate a/d conversion is performed for each channel, corresponding to each individual sensor (28). The platform was calibrated by a known weight prior to the study. Six different measures of postural control were selected: trace length, C90 area, standard deviation X and Y (std X and std Y), and amplitude X and Y (amp X and amp Y). Trace length is calculated by summing the length of straight segments connecting points that follow in a succession (28). It represents the total distance travelled by the center of pressure. C90 Area is the area of the confidence ellipse. It represents 90% of the total area covered in the medio-lateral and anterior-posterior directions (28). Std X and std Y are the standard deviation of the distance of each measurement from the mean position in each direction along the X and Y axis, respectively (28). Amp X and Amp Y are the distance between the two furthest points in each direction along the X and Y axis, respectively (28). The sampling frequency was set to 100 Hz, with a cut-off frequency of 10 Hz as recommended for these types of measurements (8), while factory presets was used for other settings (28).

### Procedures

Assessments of postural control were performed immediately before and after the 12-week period and were conducted as part of a test battery, either in the sports hall or classrooms at the respective schools. Along with the postural control test, this battery included assessments of heart rate recovery, grip strength, standing long jump, sit-and-reach flexibility, and measurements of height and weight, which will be reported elsewhere. Pupils arrived in classes of 20–30, were then divided into smaller groups, and directed to their designated testing stations. They proceeded to the next station, in a predefined order only after everyone in their group had concluded their tests at the current station. Pupils were instructed to remain seated and quiet while others were performing their tests, however, this was occasionally disregarded by some pupils. The subsequent group of pupils arrived once the current class had completed all their tests.

**Figure 1:**
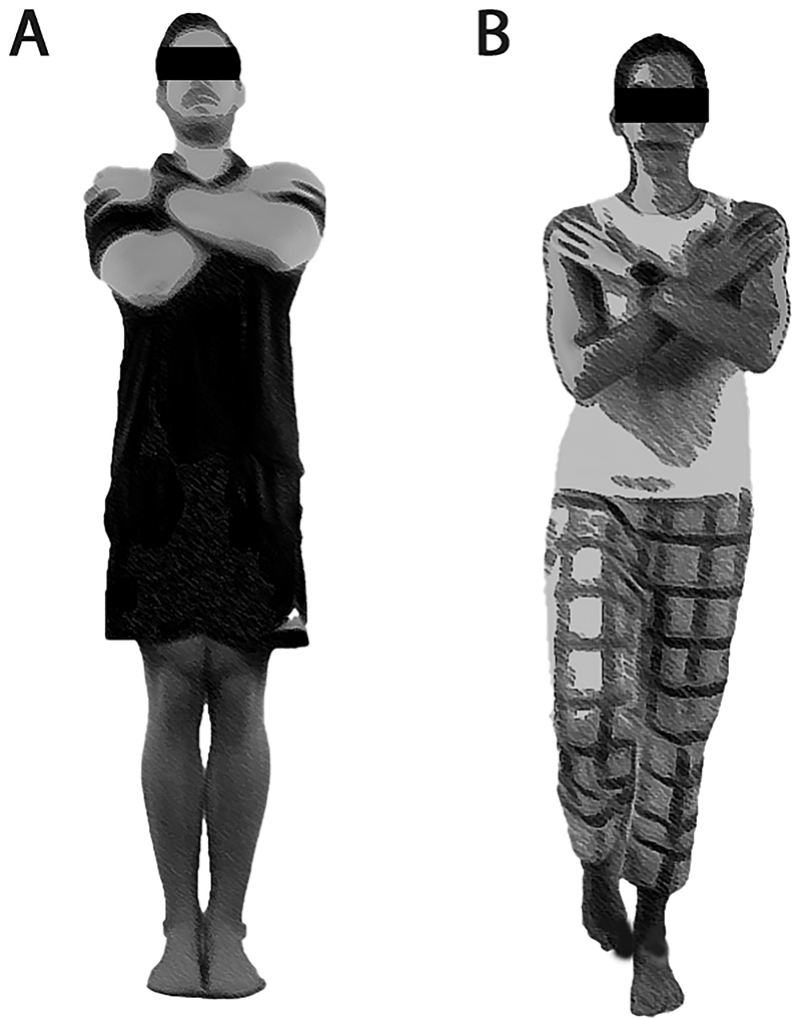
Image of body positioning in the **(A)** bipedal eyes closed condition and **(B)** the unipedal eyes open condition.

Two types of balance tests were carried out on the force platform. The first test was conducted with the participants in a bipedal eyes closed (EC) condition, while unipedal eyes open (EO) was carried out in the second test. During both tests, the participants were instructed to stand as still as possible with their arms crossed over their chest and hands resting on the opposite shoulder, following procedures in previous research (23), for 30 s. In the bipedal EC condition, the participants were asked to stand still with their feet together. In the unipedal EO condition, the participants were instructed to stand on their preferred leg for both test and retest. They were additionally instructed to start in a position with a comfortable base of support with the free leg flexed at the knee joint and the big toe placed against the medial malleolus of the standing leg (29), and to keep their gaze fixed on a mark on the opposite wall at a distance of 2 meters at eye level (8). Please refer Figure 1 for details. One measurement was carried out for each of the two tests, with a maximum of two extra attempts if one failed before 30 s had passed. The test duration was timed by the built-in timer of the force platform. A 30 s rest period was provided between each test. Both tests were performed with shoes removed. The assessor had several years of experience with these types of tests and the same assessor was used for both test and retest (20). Information was standardized and given in the same tone for all participants (20).

The force platform registered participants' weight while standing still on the platform.

Height was measured in a standing position without shoes using a portable Seca 217 (SECA GmbH, Hamburg, Germany).

Number of years from peak high velocity YPVH was calculated for boys [−7.999994 + 0.0036124 × (Age × Height)] and girls [−7.709133 + 0.0042232 × (Age × Height)] separately as recommended by Moore et al. (30).

### Analysis

A paired *t*-test was used to assess if test-retest results remained stable at a group level throughout the study and *p*-values were reported alongside means and standard deviations for all outcomes (20). Test-retest reliability was assessed by intraclass correlation coefficient (ICC) and associated 95% confidence interval (20), based on two-way mixed effects, single measurement, and absolute agreement (31). Values of ICC less than 0.5 suggest low test-retest reliability, those ranging from 0.50 to 0.75 signify moderate reliability, ICC values between 0.75 and 0.90 denote good reliability and ICC values exceeding 0.90 are indicative of excellent reliability (31). Standard error of measurement (SEm) and smallest detectable change (SDC was calculated for all postural control measures, alongside Bland-Altman plots. To assess potential associations between body characteristics and test-retest reliability, Pearson correlations were performed between height, weight, BMI, and YPHV and the square root of the sum of the square for the change from test to retest (√delta value^2^) for all postural control measures. Statistical tests were performed in SPSS version 29 (IBM Corporation, Armonk, NY, USA) and results were considered significant at the *p* < 0.05 level.

## Results

Participants (*n* = 215) were boys (44.3%), girls (53.4%), and non-binary (2.3%) aged 16–17 years. Mean height = 172.1 ± 9.2 cm, weight = 67.0 ± 14.2 kg, and BMI = 19.4 ± 3.8 kg/m^2^. YPHVwas 2.7 ± 0.4 for boys and 3.9 ± 0.5 for girls.

Based on paired *t*-tests, no significant differences were observed between the baseline and 12-week follow-up for any of the measures regardless of condition (Table 1), confirming the assumption that measures of postural control remained stable during the wash-out period.

**Table 1:** Test-retest values (mean ± SD) for the various measures in bipedal eyes closed (EC) condition and unipedal eyes open (EO) condition.

	Bipedal stance, EC			Unipedal stance, EO		
	Test	Retest	*p*	Pre-test	Re-test	*p*
Trace length (mm)	707.5 ± 210.8	694.1 ± 195.3	0.190	1,199.6 ± 412.4	1,160.0 ± 491.1	0.211
C90 area (mm^2^)	657.3 ± 344.4	678.9 ± 415.9	0.280	957.0 ± 1,282.6	975.4 ± 1,403.4	0.885
Std X (mm)	6.7 ± 2.0	6.7 ± 2.0	0.316	6.9 ± 3.9	7.0 ± 4.0	0.803
Std Y (mm)	6.7 ± 2.0	6.8 ± 2.3	0.316	8.7 ± 3.6	8.6 ± 3.7	0.816
Amp X (mm)	36.7 ± 11.6	36.3 ± 10.6	0.555	36.0 ± 16.7	36.8 ± 22.0	0.691
Amp Y (mm)	34.4 ± 9.7	34.9 ± 10.6	0.514	50.5 ± 30.7	51.4 ± 32.4	0.741

C90 area, area of the confidence ellipse; Std X, standard deviation X axis; Std Y, standard deviation Y axis; Amp X, Amplitude along the X axis; Amp Y, Amplitude along the Y axis.

The corresponding difference is indicated by *p*-values.

### Test-retest reliability

**Figure 2:**
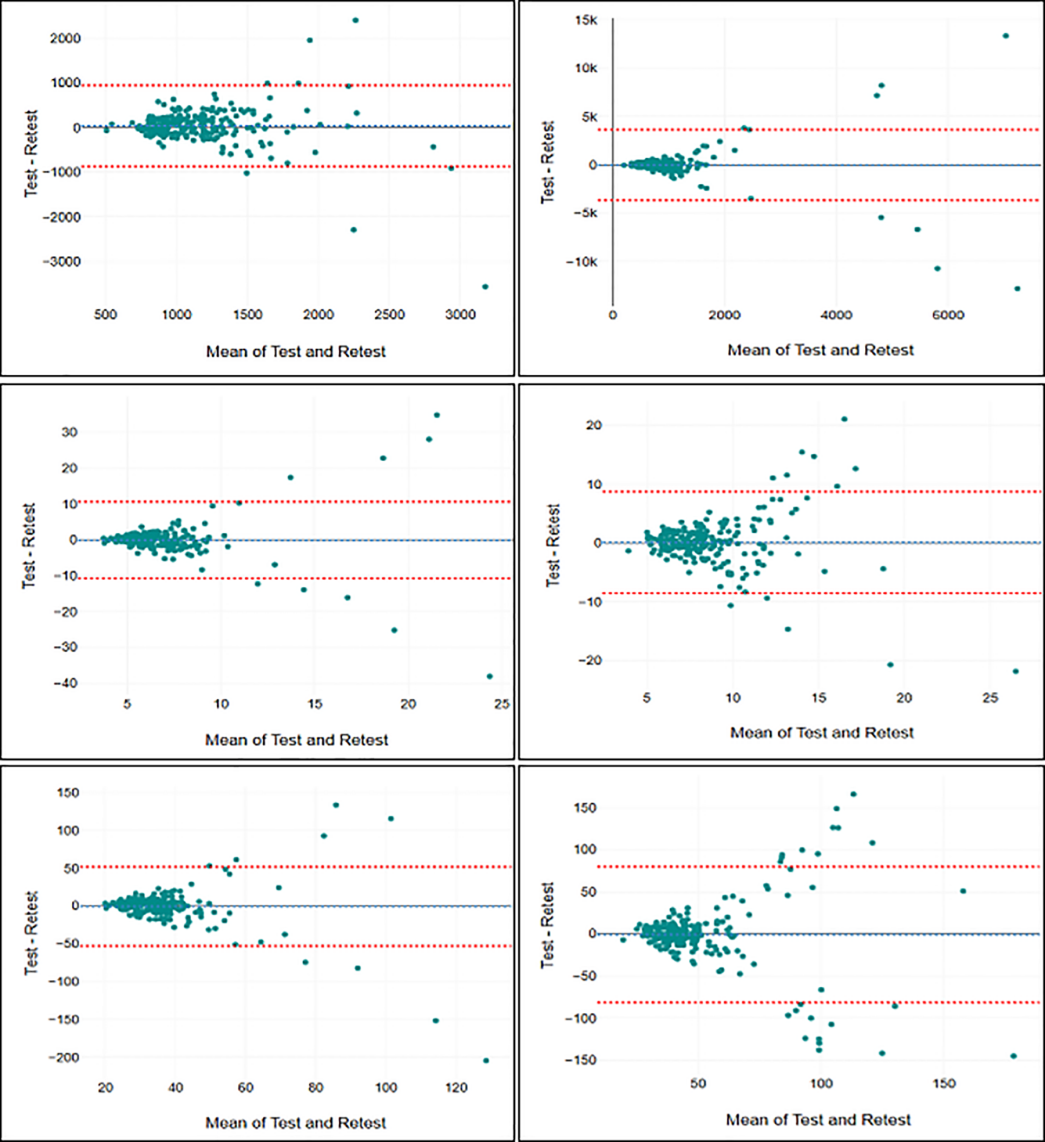
Bland-Altman plots for trace length (top left), C90 area (top right), Std X (middle left), Std Y (middle right), Amp X (bottom left), and Amp Y (bottom right) in the bipedal stance.

**Figure 3:**
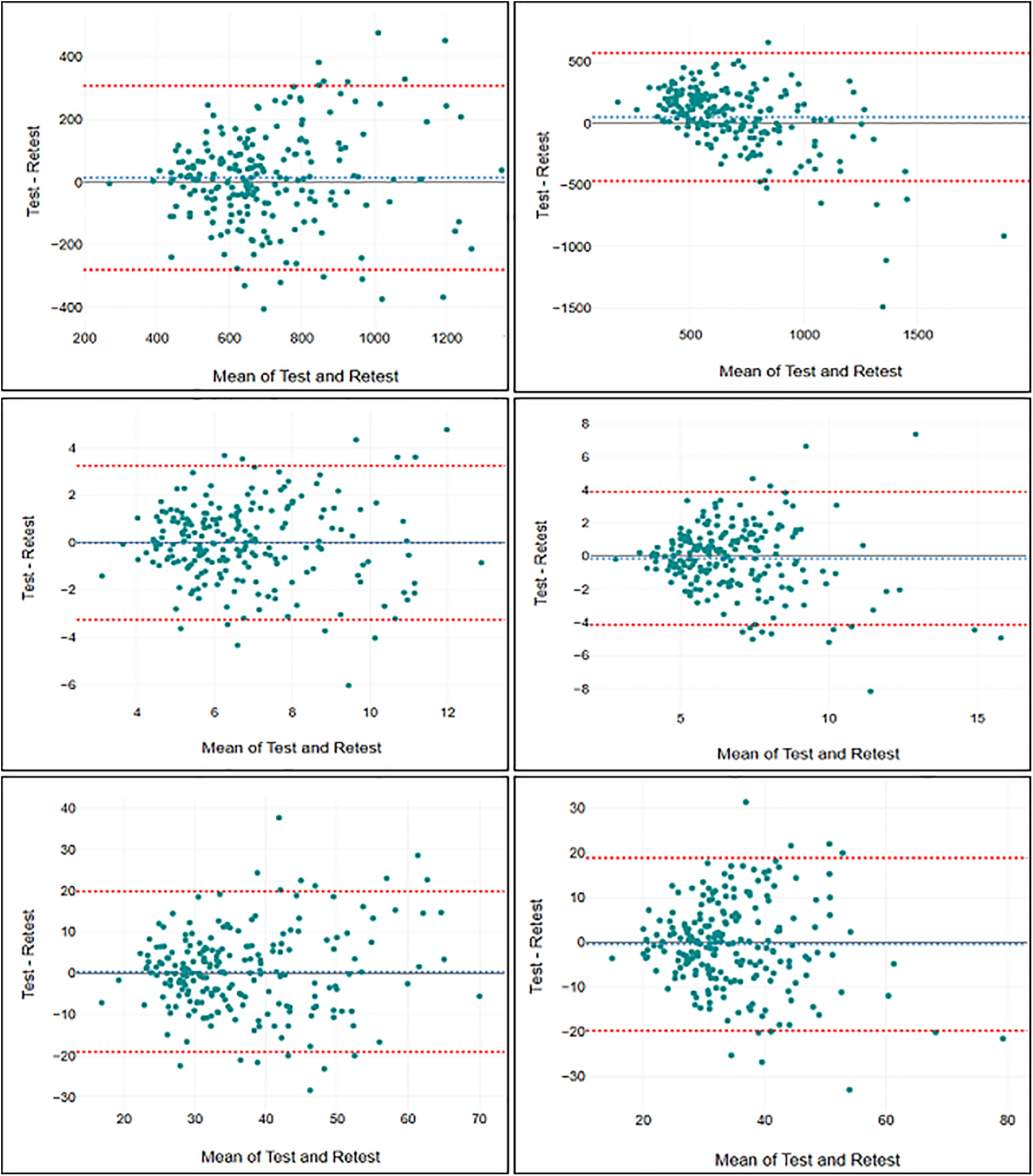
Bland-Altman plots for trace length (top left), C90 area (top right), Std X (middle left), Std Y (middle right), Amp X (bottom left), and Amp Y (bottom right) in the unipedal stance.

With regard to assessment of test-retest reliability for the bipedal eyes closed condition, the ICC values ranged from 0.53 (95% CI: 0.43–0.62) for Amp Y to 0.73 (95% CI: 0.66–0.78) for trace length (Table 2). This suggests a moderate test-retest reliability for these measures under this condition, approaching good reliability for trace length and C90 area. For the unipedal EO condition, the ICC values were generally lower, ranging from 0.03 (95% CI: −0.10–0.17) for Std X to 0.48 (95% CI: 0.37–0.57) for trace length. This indicates poor test-retest reliability for all these measures under this specific condition, although trace length was approaching moderate reliability. The accompanying SEm and SDC are reported in Table 3, while Bland-Altman plots are presented in Figures 2, 3.

**Table 2:** Intraclass correlation coefficient (ICC) with corresponding 95% confidence interval (CI) for all postural control measures.

	Bipedal stance, EC		Unipedal stance, EO	
	ICC	95% CI	ICC	95% CI
Trace length (mm)	0.73	(0.66–0.78)	0.48	(0.37–0.57)
C90 area (mm^2^)	0.71	(0.63–0.77)	0.04	(−0.09 to 0.17)
Std X (mm)	0.66	(0.57–0.73)	0.03	(−0.10 to 0.17)
Std Y (mm)	0.55	(0.45–0.63)	0.26	(0.13–0.38)
Amp X (mm)	0.60	(0.51–0.68)	0.07	(−0.07 to 0.20)
Amp Y (mm)	0.53	(0.43–0.62)	0.15	(0.02–0.28)

C90 area, area of the confidence ellipse; Std X, standard deviation X axis; Std Y, standard deviation Y axis; Amp X, Amplitude along the X axis; Amp Y, Amplitude along the Y axis.

**Table 3:** Standard error of measurement (SEm) and smallest detectable change for all postural control measures.

	Bipedal stance, EC		Unipedal stance, EO	
	SEm	SDC	SEm	SDC
Trace length (mm)	211.4	585.8	413.1	1,145.2
C90 area (mm^2^)	345.0	956.2	1,283.6	3,557.9
Std X (mm)	2.6	7.1	4.9	13.5
Std Y (mm)	2.7	7.4	4.4	12.2
Amp X (mm)	12.2	33.9	17.6	48.9
Amp Y (mm)	10.4	28.8	31.6	87.7

C90 area, area of the confidence ellipse; Std X, standard deviation X axis; Std Y, standard deviation Y axis; Amp X, Amplitude along the X axis; Amp Y, Amplitude along the Y axis.

### Correlations with change in postural control

The correlations between body characteristics (height, weight, and BMI) and the change in postural control measures (delta values) from test to retest are presented in Table 4. Weak, but significant associations were revealed between height and delta values for Std X in the bipedal EC condition and Std Y for the unipedal EO condition. No other significant associations were observed.

**Table 4:** Pearson correlation coefficients between body characteristics (height, weight, BMI, YPHV) and change in postural control (delta values) from test to retest.

	*Δ*Trace length (mm)	*Δ*C90 area (mm^2^)	*Δ*Std X (mm)	*Δ*Std Y (mm)	*Δ*Amp X (mm)	*Δ*Amp Y (mm)
Height						
Bipedal stance, EC	0.12	0.07	0.16*	0.04	0.14	0.03
Unipedal stance, EO	0.11	0.10	0.11	0.16*	0.11	0.11
Weight						
Bipedal stance, EC	0.10	0.05	0.06	−0.01	0.11	0.01
Unipedal stance, EO	−0.03	−0.06	−0.03	−0.02	−0.01	−0.01
BMI						
Bipedal stance, EC	0.07	0.07	−0.01	−0.03	0.06	−0.05
Unipedal stance, EO	−0.09	−0.11	−0.09	−0.05	−0.05	−0.03
YPHV						
Bipedal stance, EC	0.01	0.13	−0.03	0.17	−0.03	0.12
Unipedal stance, EO	−0.03	−0.06	−0.02	−0.13	0.00	−0.08

EC, eyes closed; EO, eyes open; BMI, body mass index; C90 area, area of the confidence ellipse; Std X, standard deviation X axis; Std Y, standard deviation Y axis; Amp X, Amplitude along the X axis; Amp Y, Amplitude along the Y axis; *Δ*, delta values for test-retest.

* *p* < 0.05.

## Discussion

This field-based mass testing study demonstrated that measures of postural control exhibited varying degrees of test-retest reliability, with trace length being the most reliable across both test conditions and with moderate to good reliability in the bipedal EC condition. In contrast, some of the measures revealed close to zero reliability. Interestingly, all measures exhibited greater test-retest reliability in the bipedal EC condition compared to the unipedal EO condition, suggesting the former as the more reliable for such assessments. Regarding body characteristics which were expected to impact test-retest reliability, only height was significantly correlated, albeit weakly, with the change in test-retest scores.

### Test-retest reliability of individual measures

Indeed, the limited sampling durations and the lack of averaging of results across multiple attempts in this study were expected to hamper test-retest reliability (8). Direct comparisons to previous reliability studies are challenging due to the influence of various factors, such as sampling rates, cut-off frequencies, arm positioning, trial durations, and the statistical methods employed, all of which are not consistently reported (5, 8). Ruhe et al. (8) highlighted in their review that, under lab-based conditions, postural COP can achieve an ICC of 0.75 or higher with adequate trial repetitions and sampling durations, typically 90 s with 3–5 trials. Some studies have even reported ICC's of 0.90 or above (13). However, in our study, none of the COP measures reached an ICC of 0.75, and some measures, such as C90 area and Std X in the unipedal EO condition, approached zero test-retest reliability. This highlights the importance of knowing the reliability of the measurements one intends to use in the specific testing situation and suggests that certain measures should be omitted from field-based mass-testing. Moreover, low reliability is directly associated with reduced statistical power (32), which means that studies using assessments of postural control under field-based experimental conditions should aim for a high number of participants. The results for SEm and SDC further indicate that due to high measurement error these procedures are not recommended to be used for applications at the individual level, but rather be used for comparisons of large study samples as intended. Additionally, the Bland-Altman plots indicate that the measurements included in this study has larger errors and is less reliable for participants that exhibit the lowest level of postural stability.

In contrast to the relatively low test-retest reliability for some measures, trace length showed the best test-retest reliability in both conditions in line with our hypothesis 1, achieving moderate to good reliability in the bipedal EC condition. This measure also achieved the highest ICC in the unipedal EO condition, which is consistent with previous research that identified trace length as one of the most reliable measures (5). In their review, Paillard and Noe (5) describe other measures such as C90 area, Std X and Y, and Amp X and Y as reliable as well, which was supported by Ruhe et al. (5) who claim that no single COP measurement can be singled out as the most reliable. However, there is a warning against measures that are based on a limited set of data points such as Amp X and Y (8). These two reviews mainly include studies conducted under highly controlled conditions with either longer sampling durations and/or averaging of results across multiple attempts (5, 8). When such procedures are not feasible, our results suggest that trace length is the most reliable measure considering both the bipedal EC condition and the unipedal EO condition. While other measures assess different aspects of postural control (5), our findings question their use in research without more comprehensive standardization procedures and longer or repeated measurements.

### Test-retest reliability of testing conditions

While our findings emphasize the test-retest reliability of trace length in both test conditions, there were important differences in the overall test-retest reliability of all measures across the two conditions. The bipedal EC condition showed higher test-retest reliability across all measures compared to the unipedal EO condition, in line with our hypothesis 2. This finding is also consistent with previous research, which suggests that the bipedal EC condition produces the most reliable results (8), and may be related to the fact that participants were more stable and showed higher levels of postural control during the bipedal EC condition compared to the unipedal stance with EO. Previous research further suggests that during a stable bipedal condition, postural control is less dependent on active control by the nervous system and more dependent on passive stability of the musculoskeletal system (33). This may improve reliability as the passive stability of the musculoskeletal system is expected to be less subject to temporal fluctuations compared to the ability of the nervous system to coordinate muscle activity based on an intricate sensory system. Due to the low test-retest reliability observed in the unipedal EO condition, this study questions its inclusion in field-based mass-testing. It may be a more efficient use of time to repeat the bipedal EC condition to increase reliability (8), although unipedal conditions are considered more challenging and thus more sensitive to change (27).

### Factors associated with changes in test-retest performance

In addition to understanding the overall test-retest reliability of postural control measures, it is also important to consider factors that may improve or reduce reliability, such as differences in body characteristics. Previous studies have hypothesized that body characteristics like height, weight, and BMI, known for their negative impact on postural control, might also adversely affect its reliability (8). Among the body characteristics included in this study, i.e., height, weight, and BMI, only height was found to be significantly associated with a change in postural control from test to retest, partially confirming our hypothesis 3. Neither weight nor BMI showed any significant correlation with the test-retest reliability of postural control measures. This implies that only specific body characteristics may have a detrimental effect on the reliability of these measures. One potential explanation for lower test-retest reliability among taller participants is an imbalance between the rapid growth development in this age group and the time it takes for the nervous system to adapt to the length of the body segments for optimized postural control (34). This imbalance may reduce the nervous systems' ability to accurately control posture and may lead to more variable performances. However, the associations between height and test-retest reliability were observed in only two out of the 12 measures, and both were considered weak. This suggests that taller individuals are not at a significant disadvantage in terms of reliability in our study, contrary to previous research. Chiari et al. (29) found a correlation of 0.59 between trace length and height and 0.52 between trace length and weight in an eyes closed condition similar to ours. Similarly, Hue et al. (31) reported correlations ranging from 0.16 to 0.63 between weight and various COP measures in an eyes closed condition among participants with a BMI range of 17.4–63.8 kg/m^2^ under similar conditions. The discrepancies between these studies and our findings may be attributed to differences in sampling rates, cut-off frequencies (19), and/or differences in the range of BMI among participants (35). Given these substantial discrepancies further research is essential to clarify these relationships.

### Strengths and limitations

In light of the considerations outlined in the introduction, we included many of the established recommendations for postural control and reliability research (8, 20) including a sample size of more than 200 participants, a wash-out period, standardization procedures, statistical procedures, and a detailed description of participants. Due to the nature of field-based mass-testing, we experienced some standardization issues, such as potential distractions from fellow pupils that may have diverted attention from the testing procedures. The inclusion and exclusion criteria also allowed for a diverse set of participants to be included without considering potential factors that could hamper reliability, such as visual impairments. However, the purpose of our study is to assess the test-retest reliability under such challenging conditions, i.e., without highly controlled procedures and/or time constraints. Due to this reliability study being part of a larger project an extended wash-out period was employed. This could potentially lead to real changes in postural control among some pupils. However, the *t*-test revealed no significant changes at the group level, suggesting that any impacts were minimal, if present at all.

## Conclusion

In conclusion, this study found trace length in the bipedal EC condition to be the most reliable postural control measure, while for some measures test-retest reliability estimates approached zero. The unipedal EO condition demonstrated lower test-retest reliability across all measures compared to the bipedal EC condition. Among body characteristics, only height showed a significant association with changes in postural control from test to retest. Despite the challenges inherent in field-based mass-testing, our findings highlight the need for careful selection of postural control measures, as well as thorough assessments of testing conditions in research.

## Data Availability

The raw data supporting the conclusions of this article will be made available by the authors, without undue reservation.

## References

[1] Keogh JWL O'Reilly S O'Brien E Morrison S Kavanagh JJ. Can resistance training improve upper limb postural tremor, force steadiness and dexterity in older adults? A systematic review. Sports Med. (2019) 49(8):1199–216. 10.1007/s40279-019-01141-6 31236903

[2] Nilsen DM Donato SM Halliday Pulaski K Gillen G. Standing postural control: supporting functional independence. I. In: Gillen G Nilsen DM, editors. Stroke Rehabilitation: A Function-Based Approach. 5. utg. Philadelphia, PA: Elsevier Science (2021). p. 381–412.

[3] Lin G Zhao X Wang W Wilkinson T. The relationship between forward head posture, postural control and gait: a systematic review. Gait Posture. (2022) 98:316–29. 10.1016/j.gaitpost.2022.10.008 36274469

[4] Baratto L Morasso PG Re C Spada G. A new look at posturographic analysis in the clinical context: sway-density versus other parameterization techniques. Motor Control. (2002) 6(3):246–70. 10.1123/mcj.6.3.246 12122219

[5] Paillard T Noe F. Techniques and methods for testing the postural function in healthy and pathological subjects. Biomed Res Int. (2015) 2015:891390. 10.1155/2015/891390 26640800 PMC4659957

[6] Schubert P Kirchner M. Ellipse area calculations and their applicability in posturography. Gait Posture. (2014) 39(1):518–22. 10.1016/j.gaitpost.2013.09.001 24091249

[7] Palmieri RM Ingersoll CD Stone MB Krause BA. Center-of-pressure parameters used in the assessment of postural control. J Sport Rehabil. (2002) 11(1):51–66. 10.1123/jsr.11.1.51

[8] Ruhe A Fejer R Walker B. The test–retest reliability of centre of pressure measures in bipedal static task conditions—a systematic review of the literature. Gait Posture. (2010) 32(4):436–45. 10.1016/j.gaitpost.2010.09.012 20947353

[9] Caron O. Comments about the article titled: comparison of three methods to estimate the center of mass during balance assessment, written by D. Lafond, M. Duarte, F. Prince (37 (2004) 1421–1426). J Biomech. (2005) 38(8):1737–8. author reply 1738–1740. 10.1016/j.jbiomech.2005.01.002 15958235

[10] Hof AL. Comparison of three methods to estimate the center of mass during balance assessment. J Biomech. (2005) 38(10):2134–5. 10.1016/j.jbiomech.2005.03.029 15932757

[11] Barbado D Moreside J Vera-Garcia FJ. Reliability and repetition effect of the center of pressure and kinematics parameters that characterize trunk postural control during unstable sitting test. PM&R. (2017) 9(3):219–30. 10.1016/j.pmrj.2016.08.029 27616542

[12] Corriveau H Hébert R Prince F Raîche M. Intrasession reliability of the “center of pressure minus center of mass” variable of postural control in the healthy elderly. Arch Phys Med Rehabil. (2000) 81(1):45–8. 10.1016/S0003-9993(00)90220-X 10638875

[13] Goldie PA Bach TM Evans OM. Force platform measures for evaluating postural control: reliability and validity. Arch Phys Med Rehabil. (1989) 70(7):510–7. 2742465

[14] Lafond D Corriveau H Hébert R Prince F. Intrasession reliability of center of pressure measures of postural steadiness in healthy elderly people11No commercial party having a direct financial interest in the results of the research supporting this article has or will confer a benefit upon the authors(s) or upon any organization with which the author(s) is/are associated. Arch Phys Med Rehabil. (2004) 85(6):896–901. 10.1016/j.apmr.2003.08.089 15179642

[15] Lafond D Duarte M Prince F. Comparison of three methods to estimate the center of mass during balance assessment. J Biomech. (2004) 37(9):1421–6. 10.1016/S0021-9290(03)00251-3 15275850

[16] Le Clair K Riach C. Postural stability measures: what to measure and for how long. Clin Biomech. (1996) 11(3):176–8. 10.1016/0268-0033(95)00027-5 11415618

[17] De Blasiis P Caravaggi P Fullin A Leardini A Lucariello A Perna A Postural stability and plantar pressure parameters in healthy subjects: variability, correlation analysis and differences under open and closed eye conditions. Front Bioeng Biotechnol. (2023) 11:1198120. 10.3389/fbioe.2023.1198120 37545891 PMC10399229

[18] De Blasiis P Fullin A De Girolamo CI Amata O Caravaggi P Caravelli S Posture and vision: how different distances of viewing target affect postural stability and plantar pressure parameters in healthy population. Heliyon. (2024) 10(21):e39257. 10.1016/j.heliyon.2024.e39257 39524736 PMC11550768

[19] Chiari L Rocchi L Cappello A. Stabilometric parameters are affected by anthropometry and foot placement. Clin Biomech. (2002) 17(9–10):666–77. 10.1016/S0268-0033(02)00107-9 12446163

[20] Polit DF. Getting serious about test–retest reliability: a critique of retest research and some recommendations. Qual Life Res. (2014) 23(6):1713–20. 10.1007/s11136-014-0632-9 24504622

[21] Hopkins WG. Measures of reliability in sports medicine and science. Sports Med. (2000) 30(1):1–15. 10.2165/00007256-200030010-00001 10907753

[22] Lesch KJ Lavikainen J Hyrylä V Vartiainen P Venojärvi M Karjalainen P A perturbed postural balance test using an instrumented treadmill—precision and accuracy of belt movement and test-retest reliability of balance measures. Front Sports Act Living. (2021) 3:1–12. 10.3389/fspor.2021.688993 PMC8429482 34514383

[23] Lange B Murray M Chreiteh SS Toft P Jørgensen MB Søgaard K Postural control and shoulder steadiness in F-16 pilots: a randomized controlled study. Aviat Space Environ Med. (2014) 85(4):420–5. 10.3357/ASEM.3783.2014 24754203

[24] Doyle TL Newton RU Burnett AF. Reliability of traditional and fractal dimension measures of quiet stance center of pressure in young, healthy people. Arch Phys Med Rehabil. (2005) 86(10):2034–40. 10.1016/j.apmr.2005.05.014 16213250

[25] Ghofrani M Olyaei G Talebian S Bagheri H Malmir K. Test-retest reliability of linear and nonlinear measures of postural stability during visual deprivation in healthy subjects. J Phys Ther Sci. (2017) 29(10):1766–71. 10.1589/jpts.29.1766 29184286 PMC5684007

[26] Santos BR Delisle A Larivière C Plamondon A Imbeau D. Reliability of centre of pressure summary measures of postural steadiness in healthy young adults. Gait Posture. (2008) 27(3):408–15. 10.1016/j.gaitpost.2007.05.008 17601736

[27] Paschaleri Z Arabatzi F Christou EA. Postural control in adolescent boys and girls before the age of peak height velocity: effects of task difficulty. Gait Posture. (2022) 92:461–6. 10.1016/j.gaitpost.2021.12.018 35026628

[28] HUR Labs. Balance Software Suite User Manual. (2010). Available online at: http://sd7.staattinen.fi/sites/www.hurlabs.com/files/files/balancesoftware_212_eng_manual.pdf (Accessed June 01, 2024).

[29] Barene S Holtermann A Oseland H Brekke OL Krustrup P. Effects on muscle strength, maximal jump height, flexibility and postural sway after soccer and Zumba exercise among female hospital employees: a 9-month randomised controlled trial. J Sports Sci. (2016) 34(19):1849–58. 10.1080/02640414.2016.1140906 26849477

[30] Moore SA Mckay HA Macdonald H Nettlefold L Baxter-Jones ADG Cameron N Enhancing a somatic maturity prediction model. Med Sci Sports Exerc. (2015) 47(8):1755–64. 10.1249/mss.0000000000000588 25423445

[31] Koo TK Li MY. A guideline of selecting and reporting intraclass correlation coefficients for reliability research. J Chiropr Med. (2016) 15(2):155–63. 10.1016/j.jcm.2016.02.012 27330520 PMC4913118

[32] Matheson GJ. We need to talk about reliability: making better use of test-retest studies for study design and interpretation. PeerJ. (2019) 7:e6918. 10.7717/peerj.6918 31179173 PMC6536112

[33] Henry SM Fung J Horak FB. Effect of stance width on multidirectional postural responses. J Neurophysiol. (2001) 85(2):559–70. 10.1152/jn.2001.85.2.559 11160493

[34] Bisi MC Stagni R. Development of gait motor control: what happens after a sudden increase in height during adolescence? Biomed Eng Online. (2016) 15(1):47. 10.1186/s12938-016-0159-0 27197813 PMC4874000

[35] Hue O Simoneau M Marcotte J Berrigan F Doré J Marceau P Body weight is a strong predictor of postural stability. Gait Posture. (2007) 26(1):32–8. 10.1016/j.gaitpost.2006.07.005 16931018

